# Recent advances in endoscopy 

**Published:** 2019

**Authors:** Simcha Weissman, Michael A. Sciarra, David Al-Dulaimi

**Affiliations:** 1 *Department of Medicine, Hackensack University-Palisades Medical Center, North Bergen, NJ, USA*; 2 *Division of Gastroenterology, Hackensack University-Palisades Medical Center, North Bergen, NJ, USA*; 3 *Department of Gastroenterology, South Warwickshire Foundation Trust, Warwick, UK *

*Essrani R, Hickey P, Shah H.* ***Initial Experience of a Community Gastroenterology Practice with Ultraslim Colonoscopy.*** *Cureus 2019;11(5):e4663. *

The colonoscopy remains the gold standard of care for both diagnostic and therapeutic purposes. Its success is, for the most part, hinged on its ability to reach the cecum—dubbed cecal intubation—and its adequate visualization of the colonic mucosa. Amongst multiple factors ranging from an inadequate bowel preparation to anatomical barriers, the ability to achieve cecal intubation is not always possible. Hence, with a smaller diameter and robust flexibility, the use of a smaller-caliber endoscope or an ultraslim colonoscope makes more difficult procedures a possibility. At the request of both patient and provider comfort, 13 patients underwent a colonoscopy using an ultraslim colonoscope which yielded a cecal intubation rate of 92.3%. This included Patients with a prior failed colonoscopy had successful cecal intubation. With its patient preference and reduced sedation requirements, it may be the future choice for community gastroenterologists, and/or hospital programs with patients that meet the criteria.

*Lee TJ, Siau K, Esmaily S,* *et al. ****Development of a national automated endoscopy database: The United Kingdom National Endoscopy Database (NED). ****United European Gastroenterol J 2019;7(6):798-806. *

As the quality of the endoscopy varies widely amongst endoscopists and geographical region, beginning in 2013 under the auspicious of the joint advisory group, the United Kingdom (UK) created an endoscopy database known as the national endoscopy database (NED). The goal was to have an automated uploading data system from all endoscopy centers across the UK into a centralized bank. This would quickly improve quality metrics as well as create substantial research opportunities. With this system in place, it is anticipated that endoscopists would be encouraged to compare their performance with national benchmarks. Currently, only 295 out of 529 (56%) of endoscopy services in the UK are actively uploading to NED.

Moreover, NED can only perform audits of colonoscopy information at the time of the procedure. This excludes post-procedure complications and adenoma detection rate—a globally accepted benchmark for quality. With the mandatory enforcement of NED in the coming years, the UK, and perhaps the rest of the globe (if they follow suit), will experience a paradigm shift in colonoscopy quality metrics and adenoma detection rate. 

*Di Leo G, Pascolo P, Hamadeh K, et al.* ***Gastrostomy Placement and Management in Children: A Single-Center Experience.*** *Nutrients 2019;11(7). pii: E1555.*

In patients with certain chronic conditions affecting their ability to consume an adequate caloric intake, the need to provide an alternative feeding method is paramount. The placement of a gastrostomy tube (g-tube) is one such method. In patients with neurocognitive disorders under age 18, long-term complications following g-tube placement were non-significant and outweighed by the positive effects on nutrition. Its lack of surgical complications in tandem to its impact on nutritional status makes the placement of a g-tube a novel procedure in providing nourishment to patients with chronic neurocognitive impairment.

*Khan KJ, Fergani H, Ganguli SC, et al.* ***The Benefit of Fentanyl in Effective Sedation and Quality of Upper Endoscopy: A Double-Blinded Randomized Trial of Fentanyl Added to Midazolam Versus Midazolam Alone for Sedation.*** *J Can Assoc Gastroenterol 2019;2(2):86-90.*

As many patients experience some form of anxiety while undergoing an upper gastrointestinal (GI) endoscopy optimizing sedation is of the essence for both the endoscopist and patient. Non-the-less, there are potential risks associated with over-sedation, including aspiration, transient hypoxemia, and airway obstruction. Some endoscopists use midazolam or narcotics alone, while others use a combination of the two. As there is a lack of consensus about the choice of sedative use in endoscopy discretion is up to endoscopist preference. However, the use of adding fentanyl to midazolam for sedation in outpatient upper GI endoscopy has been shown to provide superior sedation. Given this statistically significant benefit, a higher quality procedure in a shorter time can be achieved with adding fentanyl to midazolam for sedation. 

*Iwaya Y, Shimamura Y, Goda K, et al.* ***Clinical characteristics of young patients with early Barrett's neoplasia*** *. World J Gastroenterol 2019;25(24):3069-3078.*

Although esophageal adenocarcinoma is generally a disease associated with age greater than 50, many young patients do develop this malignancy. However, endoscopic screening for Barrett’s esophagus (BE), a premalignant condition for esophageal adenocarcinoma, is currently not advised in patients under the age of 50. As the diagnosis of Barrett’s-related neoplasia in younger patients has been rising in recent years, the need to begin screening these younger patients is paramount. Moreover, as younger patients are more likely to be obese and experience gastro-esophageal related symptoms, the need to consider the endoscopic screening of this population is becoming urgent. 

